# Facial Skin Microbiome: Aging-Related Changes and Exploratory Functional Associations with Host Genetic Factors, a Pilot Study

**DOI:** 10.3390/biomedicines11030684

**Published:** 2023-02-23

**Authors:** Edda Russo, Leandro Di Gloria, Matteo Cerboneschi, Serena Smeazzetto, Gian Paolo Baruzzi, Francesca Romano, Matteo Ramazzotti, Amedeo Amedei

**Affiliations:** 1 Department of Experimental and Clinical Medicine, University of Florence, 50134 Florence, Italy; 2 Department of Biomedical, Experimental and Clinical Sciences “Mario Serio” University of Florence, 50134 Florence, Italy; 3 NEXT Genomics Srl, Sesto Fiorentino, 50019 Florence, Italy; 4 Department of General Laboratory, Careggi University Hospital, 50134 Firenze, Italy

**Keywords:** microbiota, skin, aging, single nucleotide polymorphisms, genetic variants, collagen, ROS

## Abstract

In this exploratory study, we investigate the variation in the facial skin microbiome architecture through aging and their functional association with host genetic factors in a cohort of healthy women, living in the same area and without cutaneous diseases. Notably, facial skin microbiota (SM) samples were collected from a cohort of 15 healthy Caucasian females, firstly divided into three age groups (younger women aged 20–35 years old; middle aged women of 36–52 years old; and older women aged 53–68 years old). Then, the recruited cohort was divided into two groups based on their facial hydration level (dry and normal skin). The facial SM revealed a different composition in the three analyzed aging groups and between normal and dry skins. The middle-aged women also revealed functional variations associated with collagen biosynthesis and oxidative stress damage repair. Otherwise, the association between selected host SNPs (single nucleotide polymorphisms) and the facial SM profile showed significant associations, suggesting a negative correlation with collagen metabolism and ROS damage protection. Finally, the composition and functionality of the facial SM seemed to affect the aging process through the two aging-correlated pathways of host ROS damage repair and collagen metabolism. Our exploratory data could be useful for future studies characterizing the structure, function, and dynamics of the SM in the aging process to design personalized therapeutic agents focusing on potential genomic targets, microbes, and their metabolites.

## 1. Introduction

According to recent studies, skin, aside from the gastrointestinal tract, hosts the most microorganisms in the human body, with significant inter-individual differences [1,2,3]. This means that the individual’s skin health is influenced by the composition of the skin microbiota (SM). Gender, age, health condition, social contacts, interactions with the environment, and geographic location all have an impact on the richness of skin microbial populations [4,5]. In addition, by interacting with the host’s innate immune system, the SM promotes homeostasis [6]. However, specific host pathological conditions, such as immunodeficiency or skin disease, generate dramatic alterations in the skin microbiome [6]. Indeed, SM dysbiosis affects skin health and has been related to psoriasis, eczema, acne, atopic dermatitis, and other dermatological diseases [7,8,9]. Furthermore, metagenomic research on human skin has revealed that its biogeography and uniqueness influence the SM temporal dynamics, as well as its structural and functional makeup [1]. Whereas the SM associations with the host gender, geography, and numerous skin disorders have been widely examined, the link with age has been understudied.

In general, skin aging is a normal and unavoidable process characterized by structural and functional changes in skin cells, as a result of biological age, as well as external factors (e.g., exposure to ultraviolet radiation, pollution, and poor nutrition) [10]. Although biological age defines the baseline skin-aging rate, it can be difficult to distinguish between intrinsic and extrinsic causes of skin aging, such as age spots, wrinkles, sagging, loosening, and dryness. Skin aging is also affected by decreasing epidermal thickness and water content, fat emulsion, lipid content, and changes in amino acid composition [11,12]. These common skin-aging changes are thought to be a multi-factorial process that can be hastened by a variety of environmental, lifestyle, and/or socioeconomic factors. Furthermore, previous studies highlighted other fundamental intrinsic human variables that influence the density and diversity of microbes present in various host skin areas [13]. For example, some face skin characteristics, such as hydration, are known to vary across individuals and even between various parts of the skin within one individual. Nevertheless, these skin features and their interplay with microbial flora are not fully understood.

Moreover, host genetic factors influence the development of human microbial communities [14]. For example, single nucleotide polymorphisms (SNPs) in the MEFV gene, involved in inflammation, have been linked to alterations in the microbial community structure of the human gut [15], and IBD (Inflammatory Bowel Disease)-risk loci are also associated with changes in gut microbiota composition [16]. Similarly, a loss-of-function mutation in the gene FUT2, which is linked to Crohn’s disease, might affect the energy metabolism of gut microbiota [17]. A recent study analyzed the SM of monozygotic and dizygotic twins to identify the association of SM components with their host genetic factors [18]. The analysis was focused on host genes related to key dermatological conditions, including sebum production, skin humidity, pigmentation, epidermal barrier function, and hair follicle development. They found that SM diversity was significantly influenced by age and skin pigmentation. Finally, they identified one human SNP in the host FLG gene related to epidermal barrier function strongly associated with the abundance of *Corynebacterium jeikeium* [18]. The effects of microbial flora and their metabolites on other human dermatological functions such as collagen production and repair of oxidative stress damage remain poorly studied.

Starting from these premises, in this pilot study, we used an exploratory approach to investigate healthy (without diagnosed dermatological disorders) women, the age-related characteristics of the microbial community and functional pathways of the facial skin microbiome belonging to the metabolism, genetic and environmental information processing, and cellular process categories. The first aim was to assess if the microbiome might be involved in the mechanisms of skin aging by examining differences in the facial SM distribution and microbiome functional pathways, in three groups of women living in the same area. In the second part, we sought to determine whether observed differences in the nature and diversity of the facial SM correlate with skin hydration level.

Finally, we observed if a specific microbial profile is associated with host genetic variations, evaluating the correlation between SM taxa and SNPs (considering genes related to key dermatological conditions). Significant functional associations of the skin microbiome with host collagen and oxidative stress pathways were observed for the first time, highlighting the need to obtain a deeper understanding via future in vitro and in vivo studies.

## 2. Methods

### 2.1. Patients Recruitment and Facial Skin Sampling

We enrolled a cohort of 15 healthy female Caucasian volunteers (mean age: 44.5 ± 14 years) without diagnosed dermatological disorders or antibiotic treatment for the past 6 months (Table 1). These Italian women were divided into three age-related groups, namely, younger women aged 20–35 years old, middle-aged women of 36–52 years old, and older women aged 53–68 years old. In particular, the range of the older women group was defined based on the presence of the menopausal condition. Each participant was advised not to wash her face with soap or take a bath at least 12 h before arrival at the sample collection site. On arrival, their faces were washed with sterile water (Milli-Q water) and they were put in a controlled environment at 24 °C and relative humidity of 45% for a minimum period of 4 h before sample collection. In this way, we provided sufficient time for the resident facial skin microflora and the levels of skin health parameters such as hydration to regain their individuality. Using a corneometer (measures hydration, Courage + Khazaka electronic GmbH, Köln, Germany), readings on levels of hydration from the forehead and cheek regions were taken on each participant to measure hydration levels.

The moisture-related skin types were determined as follows: dry skin was characterized by corneometer units less than 45 and normal skin higher than 45 A.U. (arbitrary units) [19].

A questionnaire with information on lifestyle, location, sun exposure, facial make up, use of sun protection, and routine skincare was administered.

Skin swabs were collected from facial regions, excluding lips and nose, using moistened sterile pads (2 × 2 cm) for 10 s each. Buffer used for sample collection was 1X Phosphate Buffer Saline (PBS) pH 7.0 + Tween 80 (0.5%). We ensured that the volunteers covered their entire face (except for the region around the mouth and nose) with moist sterile pads. The sterile pads were then put in sterile tubes containing 10mL of 1X PBS buffer. The tube was vortexed for 5 min to ensure that all microbes were suspended in the buffer. The supernatant was then centrifuged for 15 min at 15,000× *g* and the microbial pellet was separated. Negative controls were prepared by exposing a swab pad to the sample collection room for 10 min and processed as described above.

### 2.2. Microbial DNA Extraction and Next Generation Sequencing (NGS)

Genomic DNA was isolated from the microbial pellets following the manufacturer’s protocol and a Microbiome DNA Isolation Kit (Norgen Bioteck Corp. Thorold, ON, Canada). The quality and quantity of extracted DNA were assessed using the Qubit Fluorometer (Thermo Fisher Scientific, Waltham, Massachusetts, USA) and then genomic DNA was stored at −20 °C. The mean DNA yield was 33.45 ± 16.22 ng and the UV absorbance 260/280 ratio was greater than 1.86 (260/230 ratio over 2.11). Extracted DNA samples were sent to NEXT Genomics (Sesto Fiorentino, Italy) where amplicons of the variable V3–V4 region of the bacterial 16s rRNA gene, delimited through primers 341F and 805R, were sequenced in paired-end (2 × 250 cycles) on the Illumina MiSeq platform, according to the Illumina 16S Metagenomic Sequencing Library Preparation protocol [20,21].

### 2.3. Bioinformatics for Microbial Community Analysis

Raw Illumina NGS sequences were processed using QIIME2 2021.4. Briefly, sequencing primers were removed with Cutadapt and DADA2 commands were used to perform paired-end read merging, filtering, and chimera removal steps after trimming nucleotides from forward and reverse reads based on the quality profiles (--p-trunc-len-f 223 and --p-trunc-len-r 219). Hence, ASVs (amplicon sequence variants) were generated and the V-search tool was used for taxonomic assignment through the SILVA database (release 138) as a reference with a 0.99 identity threshold (Table 2).

Statistical analyses on the bacterial communities were performed in R 4.1 (R Core Team, 2014, Vienna, Austria) with the help of the packages phyloseq 1.36.0 [22], DESeq2 1.32.0 [23] and other packages satisfying their dependencies, in particular, vegan 2.5-7. Packages ggplot2 3.3.5, dendextend 1.15.1 [24], pca3d 0.10.2, and ggpubr 0.4.0 were used to plot data and results. Rarefaction analysis of ASVs was performed using the vegan function rare curve (step 100 reads) and further processed to highlight saturated samples (arbitrarily defined as saturated samples with a final slope in the rarefaction curve with an increment in ASV number per reads <1e-5). Principal coordinate analysis (PCoA) with the Bray–Curtis similarity index and hierarchical cluster analysis with Euclidean distance were performed on per cent normalized count data of ASVs of each sample, adjusted with square root transformation taking into account the characteristics of microbial data sets. [1]. Observed Richness, Shannon, and Evenness indices were used to estimate bacterial diversity in each sample using the function estimate_richness from phyloseq. The evenness index was calculated using the formula E = S/log(R), where S is the Shannon diversity index and R is the number of ASVs in the sample. Differences in all indices were tested using the Wilcoxon–Mann–Whitney test (for dry skin comparison) or the Kruskal–Wallis test (for age comparison). Beta diversity was calculated through PERMANOVA [25] analysis using the function adonis (9999 permutations) in vegan, with the Bray–Curtis distance of proportional genus data of each sample. Pairwise beta dispersion was calculated using the functions betadisper and permutest (9999 permutations) of the vegan package with the Bray–Curtis distance of proportional genus data of each sample.

The differential analysis of abundance was performed with DESeq2 on raw data at the different taxonomic ranks (created using the tax_glom function in phyloseq) after performing a minimal pre-filtering to keep only taxa with a global abundance of at least 10. PICRUSt2 [26] with the SEPP algorithm was used to predict functional abundances based on the KO (KEGG ORTHOLOGY) database, and then LEFse (Linear discriminant analysis Effect Size) [27] on the Galaxy platform was used to calculate significant LDA after per-sample normalization of the sum of the values to 1M as suggested on the Galaxy site [28].

### 2.4. SNPs Genotyping

For SNP (single nucleotide polymorphism) genotyping, nucleic acids were obtained using the Oragene OG-500 (DNA Genotek Inc, Ottawa, ON, Canada) self-collection saliva kits collected from the participants’ homes while respecting distancing and quarantine restrictions. Human DNA was extracted from 1 mL of saliva using a Puregene DNA Purification kit (according to the manufacturer’s protocol) and genome-wide genotyping was performed using 200 ng of genomic DNA at NEXT Genomics (Sesto Fiorentino, Italy). The Illumina Infinium Global Screening Array (GSA) v3-MD (Illumina, San Diego, CA, USA) including 700,625 genomic markers was used and processed according to the manufacturer’s specifications. BeadChips were scanned using the Illumina iScan Reader. Genes of interest were selected based on the DisGenet Skin Wrinkling data set (CUI: C0037301); in detail: COG (component of oligomeric golgi complex 5), TALDO1 (transaldolase 1), ALDH18A1 (aldehyde dehydrogenase 18 family member A1), PIK3R1 (phosphoinositide-3-kinase regulatory subunit 1), LMNA (lamin A/C), KCNJ6 (potassium inwardly rectifying channel subfamily J member 6), EFEMP2 (EGF containing fibulin extracellular matrix protein 2), SLC25A24 (solute carrier family 25 member 24), ELN (elastin), POLR3A (RNA polymerase III subunit A), FBLN5 (fibulin 5), and LTBP4 (latent transforming growth factor beta binding protein 4). Then, information about which related SNPs are linked to missense or non-stop mutation, depending on having a major or minor allele, were collected through Illumina Infinium support files called “Gene annotation file” (https://support.illumina.com/downloads/infinium-global-screening-array-v3-0-support-files.html, accessed on 20 February 2023) and enriched by the Ensembl Variation 104 database (Human Short Variants data set) using the R package biomaRt 2.48.3. Because minor alleles are more commonly found to be linked to diseases [29], SNPs with minor alleles were then considered and scored as 1 if in heterozygosity or as 2 if in homozygosity, in each sample.

### 2.5. Data Availability Statement

The microbial-related data (raw reads, ASV tables, and taxonomic assignments) are freely available at the NCBI Gene Expression Omnibus under the series accession GSE225848 and the analysis script is available at https://github.com/matteoramazzotti/papers/skin2021 (accessed on 20 February 2023).

## 3. Results

### 3.1. Different Facial Skin Microbial Signatures Related to Age

The facial SM of 15 healthy women was analyzed by 16S rRNA gene amplicon sequencing. We obtained a total of 1,219,280 reads and, after all the steps of pre-processing (pair merging, trimming, quality filtering, and chimera detection), 947,090 (77%) were available for further analysis (Supplementary Materials Table S1, Figure S1).

**Figure 1:**
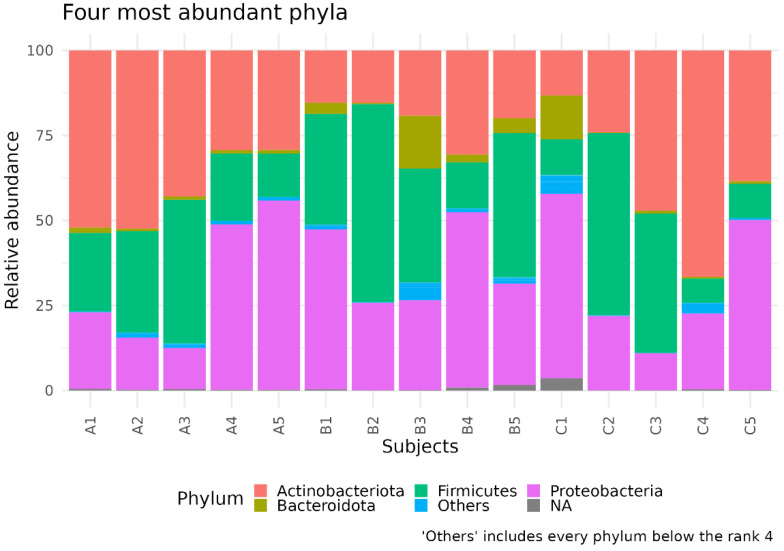
Taxonomic composition of facial skin samples. The stacked bar plot shows the relative abundance of the four more abundant bacterial phyla in each sample. The “Others” group contains phyla with ranks below five.

The analysis of the taxonomic composition revealed that more than 97% of the sequences were classified into four phyla: *Proteobacteria* (32.91%), *Firmicutes* (28.69%), *Actinobacteria* (33.07%), and *Bacteroidetes* (3.08%) (Figure 1). As the facial skin characteristics change during age [30], we wondered if skin age-dependent variations might be mirrored in the respective microbiota composition. So, we divided the enrolled female into three groups, notably subjects aged 25–35 (Group A), 36–52 (Group B), and 53–68 (Group C) years old.

**Figure 2:**
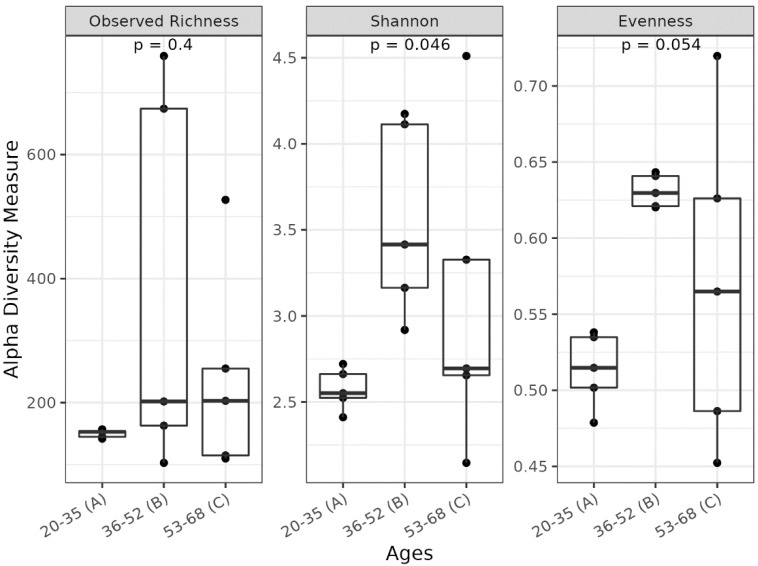
Boxplots reporting alpha diversity indices (Observed Richness, Shannon, and Evenness indices) in groups A, B, C. *p*-values less than 0.05 were considered statistically significant.

**Figure 3:**
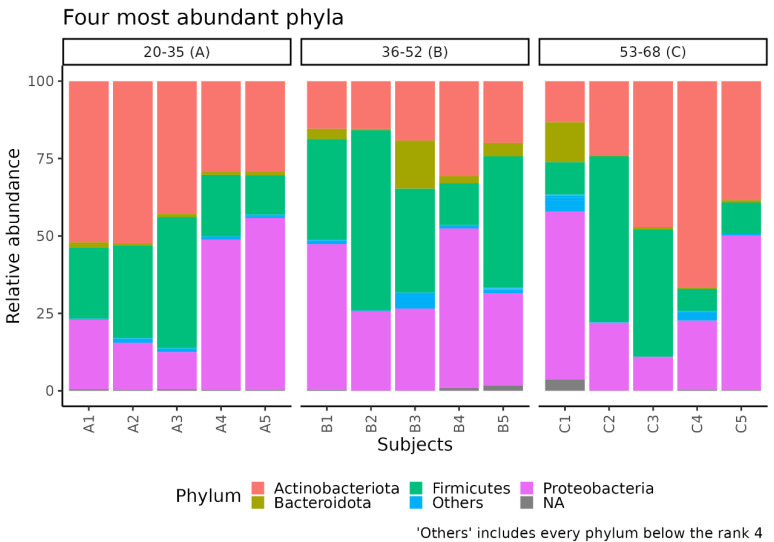
Taxonomic composition of facial skin samples in age groups A, B, and C. The stacked bar plot shows the relative abundance of the four more abundant bacterial phyla in each sample. The “Others” group contains phyla with ranks below five.

Comparing the three groups, we observed significant alterations in the SM profile. In detail, the Shannon index (*p* = 0.046) indicated a general difference in the alpha diversity of facial skin samples. Moreover, group B showed a higher Shannon index compared to that of group A (p.adj = 0.040), indicating differences in microbial variability and abundance (Figure 2B). In addition, a trend (*p* = 0.052) in the Evenness index suggests that ASVs may be more homogeneously distributed in group B (Figure 2C). Regarding the relative abundance of the most represented microbial phyla, the group B samples, which showed the highest alpha diversity value compared to groups A and C (Figure 2B), seem to have a distinct pattern of microbial distribution (Figure 3), with a wide distribution of taxa belonging to the major phyla previously indicated (notably, *Proteobacteria*, *Bacteroidetes*, and *Firmicutes*).

**Figure 4:**
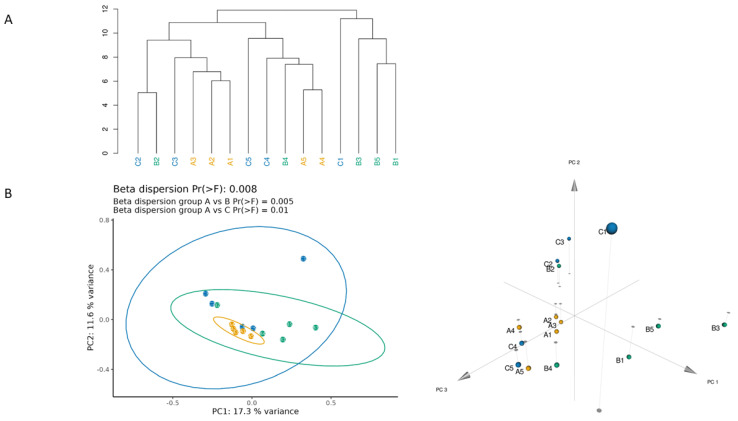
Multivariate representations of the entire sample set. (**A**) Complete hierarchical clustering based on Euclidean distance of square root transformed percent abundance of identified ASVs and (**B**) principal coordinate analysis using Bray-Curtis dissimilarity as a distance metric on square root transformed percent abundance of identified ASVs showing permuted *p*−value of general β dispersion and pairwise β dispersion of group A compared to groups B and C. Ellipses drawn around each group delineate the 95% confidence interval of the centroid position. Group A is plotted in yellow, Group B is in green, and Group C is in blue.

To investigate the similarity among subjects’ abundance profiles, we performed cluster analysis on normalized ASV counts. The hierarchical clustering suggested that the younger subjects of group A (in detail, A1, A2, A3) have a more similar microbiota composition (Figure 4A). This result was confirmed by the principal coordinate analysis (PCoA), which also displayed a significant difference in the general beta dispersion (permuted *p*-value = 0.008) and, specifically, a significantly lower beta dispersion of group A with respect to both groups B and C (permuted *p*-value = 0.005 and 0.01, respectively) (Figure 4B).

**Figure 5:**
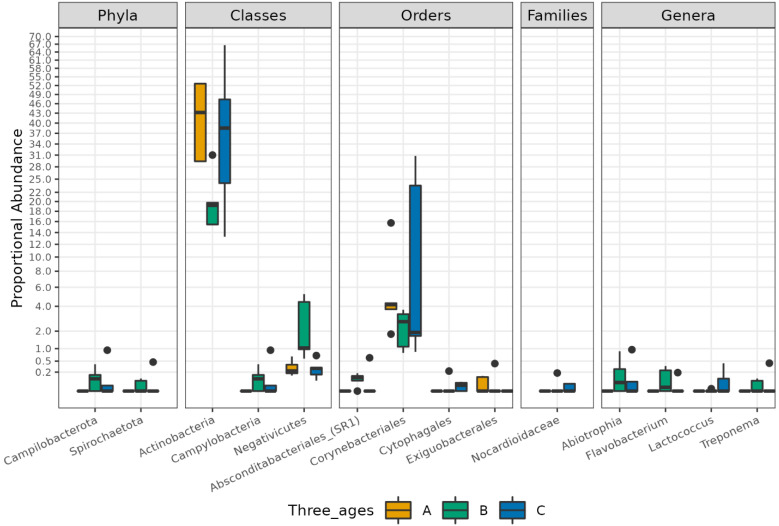
Boxplot showing the results of taxa differential abundance analysis between the three groups of subjects. Y axis has been scaled to improve the readability of values. All results have an adjusted *p*-value < 0.05.

Finally, the comparison of the abundance of a single ASV revealed significant differences between the three sample groups (adj.p < 0.05, abs (logFC)> = 1). In particular, the phyla *Campilobacteriota* and *Spirochaetota*, the class of *Campylobacteria Negativicus,* the order of *Absconditabacteriales_SR1*, and the genera of *Abiotrophia*, *Flavobacterium*, and *Treponema* were significantly higher in B group samples compared to those of groups A or C. The class of *Actinobacteria*, the order of *Corynebacteriales*, the family of *Nocardioidaceae*, and the genus of *Lactococcus* were significantly more abundant in group C compared to groups A or B, while the family of *Exiguobacteriales* was more abundant in group A compared to the B and C groups (Supplementary Materials Table S2). Graphical results of the differential analysis at all taxonomic ranks are available in Figure 5.

### 3.2. Functional Profiles of Age-Related Facial Skin Microbiota

**Figure 6:**
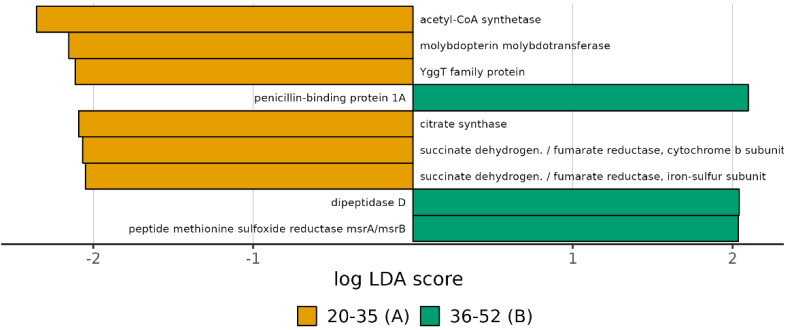
Computed LDA scores of the significantly different functions in A and B groups. Negative LDA scores (yellow) are enriched in the A group while positive LDA scores (green) are enriched in the B group.

**Figure 7:**
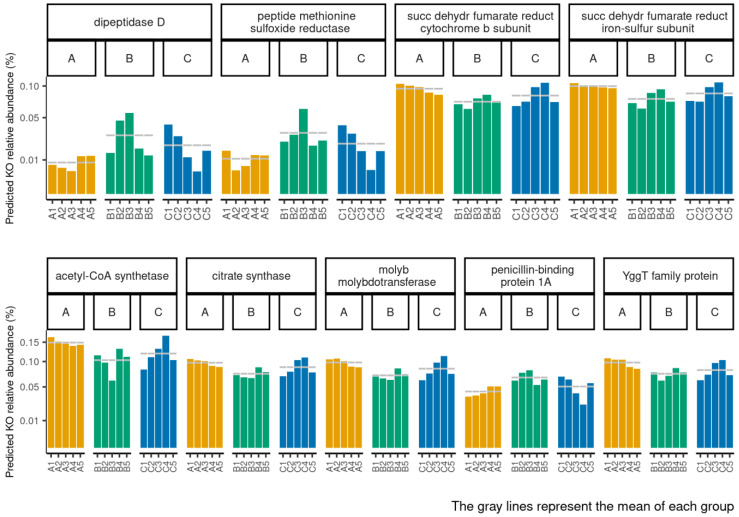
The abundance of each significantly different function in groups A, B, or C within each sample inferred by PICRUSt2. The grey lines represent the mean of each group.

The functional metagenomics contents inferred using PICRUSt2 analysis were examined to better understand how the bacterial functional profiles differed between the three age groups. We performed functional analyses through PICRUST2, including pathways involved in microbial gene functions belonging to the metabolism, genetic information processing, environmental information processing, and cellular process categories. We observed particular functional profiles associated with potentially expressed microbial genes in groups A and B. Among the functional pathways belonging to all of the categories, those predominantly found in groups A and B were identified. In particular, group B was positively associated with K05366, K01270, and K12267, while group A was associated with K00240, K00241, K01647, K02221, K03750, and K01895 (Figure 6 and Supplementary Materials Table S3). The abundance of each function within each sample and the relative mean of each group are shown in Figure 7. In particular, we observed a large proportion of the functions associated with carbohydrate, amino acid, and nucleotide metabolism.

### 3.3. Different Microbial Signatures Depend on the Hydration Level

**Figure 8:**
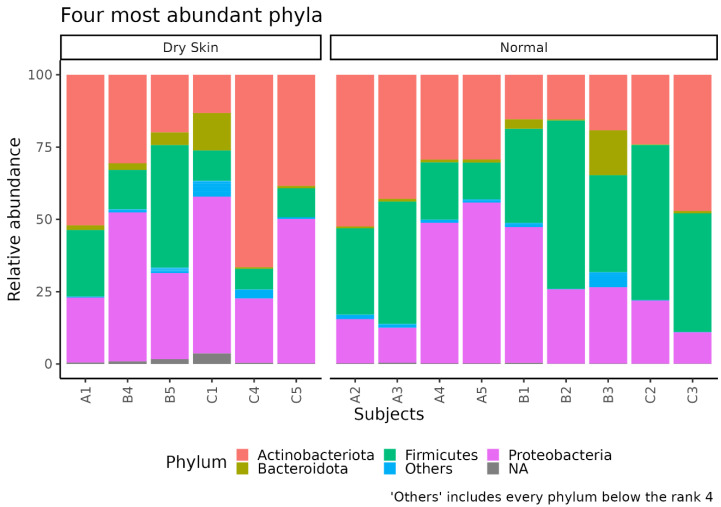
Taxonomic composition of facial skin samples in dry skin and normal skin groups. The stacked bar plot shows the relative abundance of the four more abundant bacterial phyla in each sample. The “Others” group contains phyla with rank below five.

**Figure 9:**
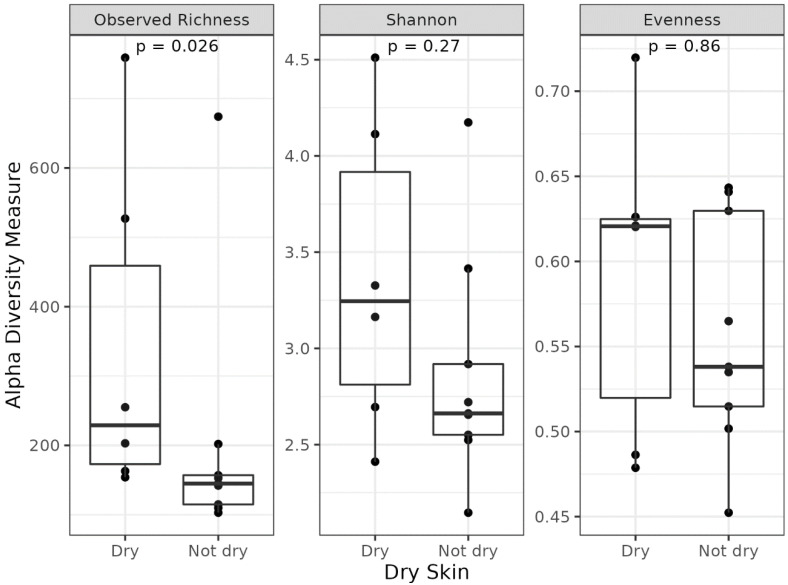
Boxplots reporting alpha diversity indices (Observed Richness, Shannon, and Evenness indices) in dry skin and normal skin groups. *P*−values less than 0.05 were considered statistically significant.

In this pilot study, we focused only on host skin characteristics such as hydration, since we considered smoke and sun exposure as environmental factors. So, to better correlate the facial microbiota with individual skin characteristics, we divided our cohort into two groups, namely, women with dry and normal skin (Figure 8). In particular, the alpha diversity of samples displayed differences for the “observed richness index” (*p* < 0.026), indicating differences in taxa richness (Figure 9).

Finally, the abundance comparison of a single ASV revealed significant microbiota alterations between the two sample groups (adj.p < 0.05, abs (logFC)> = 1).

**Figure 10:**
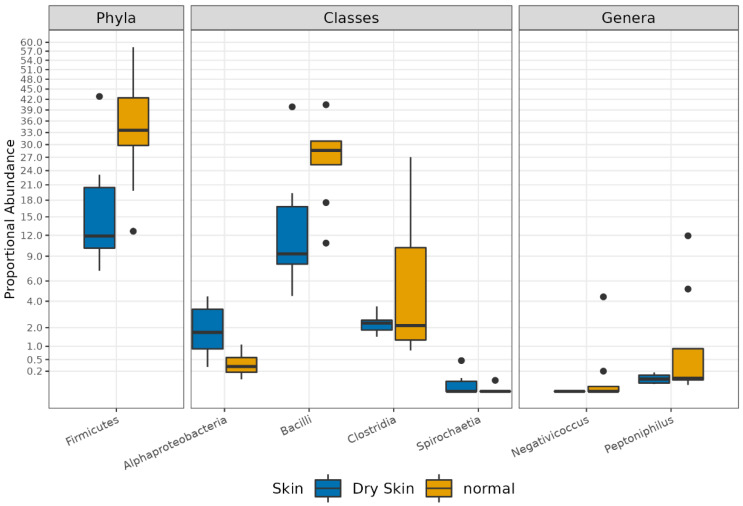
Boxplot showing the results of taxa differential abundance analysis between the dry skin and normal skin groups. Y axis has been scaled to improve the readability of values. All results have an adjusted *p*-value < 0.05.

In detail, the phylum *Firmicutes*, the class of *Clostridia*, and the genera of *Negativicoccus* and *Peptoniphylus* were significantly higher in normal skin group samples compared with the dry skin group. On the contrary, the classes of *Alphaproteobacteria* and *Spirochetia* were significantly more abundant in dry skin compared to the normal group (Supplementary Materials Table S4 and Figure 10).

### 3.4. Functional Profiles of Hydration-Related Facial Skin Microbiota

**Figure 11:**
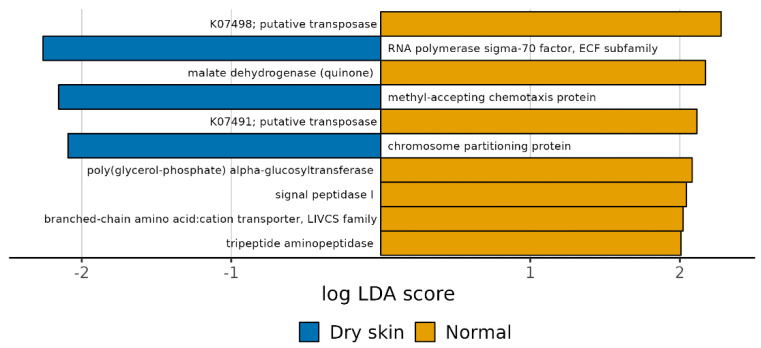
Computed LDA scores of the significantly different functions in normal and dry skin groups. Negative LDA scores (blue) are enriched in the dry skin group while positive LDA scores (yellow) are enriched in the normal group.

**Figure 12:**
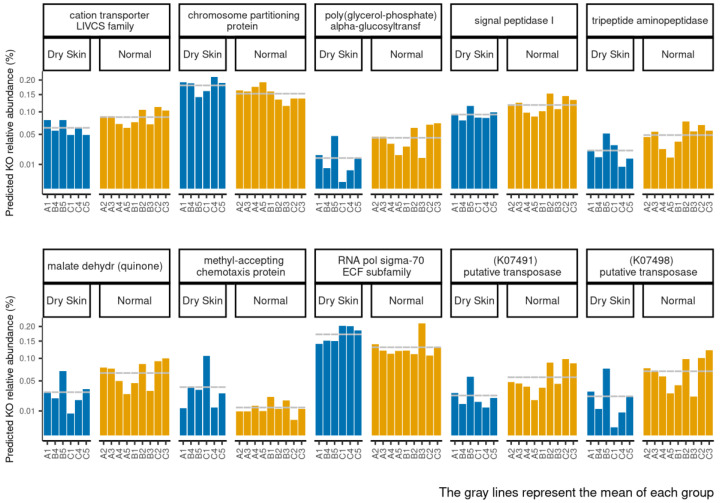
Abundance of each significantly different function in normal and dry skin groups within each sample inferred by PICRUSt2. The grey lines represent the mean of each group.

We observed particular pathways associated with expressed genes belonging to dry and normal skin groups. In particular, the dry skin group was positively associated with K03088, K03406, and K03496, while the normal skin group was negatively associated with K01258, K03311, K03100 K00712, K07491, K07498, and K00116 (Figure 11 and Supplementary Materials Table S5). The sample abundance and the group mean of each PICRUST functional profile within each sample are shown in Figure 12.

### 3.5. Genetic Association of Facial Skin Microbiota

**Figure 13:**
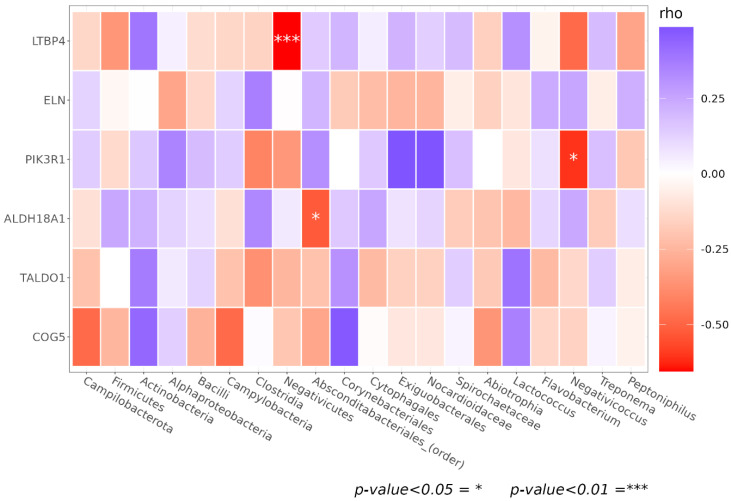
Heatmap of Spearman correlation values between minor alleles in selected SNPs of interest genes (rows) and taxa showing different abundance results in DESeq2 analyses (columns). Genes with any minor allele of relative SNP counted are not reported. *p*-values lower than 0.05 are marked with an asterisk.

Finally, we explored associations between the SM composition and host genetic variation. For this investigation, we focused on SNPs in a pre-established panel of select host genes (see Methods/SNPs genotyping section). Among the host genes, we manually selected those related to the dermatologic condition of dry skin. Spearman’s correlation was derived between the significantly changed taxa and the number of minor alleles on selected SNPs (Figure 13). We observed negative correlations between *Negativicutes* and three *LTB4* (latent transforming growth factor beta binding protein 4) SNPs (rho = −0.55 and *p*-value = 0.032), *Absconditabacteriales*_SR1 and one *ALDH18A1* (Aldehyde Dehydrogenase 18 Family Member A1) SNP (rho = −0.52 and *p*-value= 0.045), and *Negativicoccus* and one *PIK3R1* (Phosphatidylinositol 3-kinase regulatory subunit alpha) SNP (rho = −0.60 and *p*-value= 0.016), as reported in Table 3.

In detail, *LTB4* (latent transforming growth factor beta binding protein 4) is a key regulator of transforming growth factor beta (TGFB1, TGFB2, and TGFB3) which controls TGF-beta activation by maintaining it in a latent state during storage in extracellular space. Its biological function is associated with the assembly of the extracellular matrix fibers (consisting mainly of proteins, especially collagen, and glycosaminoglycans, mostly proteoglycans) that enable the matrix to recoil after transient stretching [31,32]. In addition, the proteins of the extracellular matrix provide essential physical scaffolding for the cellular constituents and can also initiate crucial biochemical and biomechanical cues required for tissue morphogenesis, differentiation, and homeostasis. *ALDH18A1* (aldehyde dehydrogenase 18 family member A1) is a member of the aldehyde dehydrogenase family and encodes a bifunctional ATP- and NADPH-dependent mitochondrial enzyme with both gamma-glutamyl kinase and gamma-glutamyl phosphate reductase activities. The encoded protein catalyzes the reduction of glutamate to delta1-pyrroline-5-carboxylate, a critical step in the de novo biosynthesis of proline, ornithine, and arginine. *PIK3R1* Phosphatidylinositol 3-kinase phosphorylates the inositol ring of phosphatidylinositol at the 3-prime position. This gene encodes the regulatory subunit. Phosphatidylinositol 3-kinase plays an important role in the metabolic insulin actions and its biological function has been related to the cellular response to UV, the epidermal growth factor receptor signaling pathway, and the negative regulation of cell-matrix adhesion.

## 4. Discussion

The skin microbiome plays an important role in preventing invading pathogens, educating the host immune system, and breaking down natural products, similar to the gut microbiome [33]. However, research on the skin microbiome is not as advanced as research on gut microbes, because the comparatively highly open skin microenvironment leads to large differences in the flora between individuals.

In this pilot study, we analyzed the facial SM composition of 15 healthy women and, in agreement with previous data, the most abundant phyla detected were *Proteobacteria* (the most representative), *Firmicutes*, *Actinobacteria*, and *Bacteroidetes* [34,35].

To profile the distribution of facial SM according to age, several studies (mostly from Asia), in contrast with ours and a few others [36], usually compared two groups, composed of younger and older subjects, with an average age range of over 20 years [37,38,39].

We added a group of middle-aged women to obtain information about the gradual variation in SM composition over time, considering a range of age with an intermediate process of aging. Comparing the three age-related groups, we observed significant alterations in microbiota profile; in particular, the middle-aged group (36–52 years) exhibited a higher alpha diversity, indicating differences in microbial variability and abundance. In addition, this group seems to have a distinct pattern of microbial organization, with a wide distribution of microbial taxa belonging to *Proteobacteria*, *Bacteroidetes*, and *Firmicutes*. Although the major microbial compositions were similar, the alpha diversity result seems to contrast with the previously mentioned studies [37,38]. In detail, Shibagaki et al. [37] and Kim et al. [39] observed that the alpha diversity was significantly higher in the elderly; in contrast, Kim et al. [38] reported higher alpha diversity in the younger group. These differences might be explained by the SM development, which can be significantly influenced by the urban and living environments, particularly the individual residential environment and lifestyle [5]. Intriguingly, we also observed that subjects A5, B4, and C5, belonging to different age groups, have a similar skin microbiota composition; this was also the case for B2 and C2. This preliminary result suggests that aging may not be the only explanatory factor that should be considered in a fine microbiota evaluation. Indeed, it was also reported that the skin microbiome differs depending on the ethnic race, which may be because endogenous (immune tone, genetic characters, and skin properties) and exogenous (e.g., foods and lifestyles) factors are different depending on ethnicity [40].

Moreover, the middle-aged group reported significantly higher levels of minor phyla such as *Campilobacterota* and *Spirochaetota*. Interestingly, in a recent study of SM*, Campilobacterota,* composed of Gram-negative bacteria, was associated with Rosacea, a common skin condition with blushing and visible blood vessels on the face [41]. The middle-aged subjects also displayed higher levels of *Campylobacteria* and *Negativicus* classes. Intriguingly, *Negativicutes* represent a particularly little-explored *Firmicutes* class, and are characterized as Gram-positive bacteria with an unusual cell wall that inhabits a wide variety of niches, including the skin and intestines [42]. Additionally, the predicted metagenomic pathway analysis of the middle-aged SM profile displayed functional association with Dipeptidase D (pepD) and peptide methionine sulfoxide reductase msrA/msrB (mrsAB). Notably, pepD is an enzyme that splits dipeptides with a prolyl or hydroxyprolyl residue in the C-terminal position. It plays an important role in collagen metabolism because of the high level of amino acids in collagen. Collagen is an essential scaffold protein that gives smoothness and elasticity to the skin, and is associated with facial wrinkles and texture, but its production declines with age [43]. Changes in the quantity, structure, and distribution of collagens in tissues may affect cell signaling, metabolism, and function. Several pieces of evidence suggest that prolidase activity may be a step-limiting factor in the regulation of collagen biosynthesis [44]. On the other hand, mrsAB is also produced by *Campylobacterales,* an order belonging to the *Camplilobacteria* class that was found to be augmented in the middle-aged group. This molecule has an important function as a repair enzyme for proteins that have been inactivated by oxidation. Indeed, reactive oxygen species (ROS) accumulate over time and are the main contributor to the aging process [45]. Different studies demonstrated a potential involvement of microbiome components in oxidative stress reactions (as reported in [46]). Our functional result might indicate that the increase in specific skin microbial taxa that produce ROS-damage-repairing enzymes may affect the aging process in middle-aged groups. However, to date we cannot know to what extent. Although these functional differences reported for the medium-aged group were not experimentally confirmed in our study, the predicted metagenomic pathways could provide meaningful information about the bacterial role in relating skin aging to the microbiome. Notably, if this hypothetic scenario is confirmed by in vitro and in vivo studies, the remodeling of the facial microbiome with specific microbial taxa producing ROS-damage-repairing enzymes could represent a new challenging concept in the design of personal anti-aging treatments.

Concerning the younger group (20–35 years), curiously it displayed an increase in *Exiguobacteriales*, a family found in various types of plants [47]. The principal microbiome functional variations observed in this group were associated with carbohydrate metabolism (citrate synthase) and acetyl-CoA synthetase, which catalyzes the synthesis of acetyl-CoA from short-chain fatty acids (SCFA) [48], the main metabolites produced by microbiota. Indeed, the metabolites produced in the skin play an essential role in host–microorganism interactions and their production is greatly influenced by our environment and behavior [49,50,51]. Interestingly, a very recent report on a model organism indicates acetyl–CoA as a critical mitochondrial signal to regulate aging through the chromatin remodeling and histone deacetylase complex [52].

In accordance with some studies [38], but in contrast with others [39], the SM composition of the older group (53–68 years) displayed a higher abundance of the *Actinobacteria* class.

It is known that SM modifications are accompanied by changes in individual skin conditions and physiology. Previous studies characterized the microbiota signatures from different skin sites, reporting considerable topographical and temporal variance across dry, moist, and sebaceous conditions [1]. The hydration level in the surface layer of the human skin, the stratum corneum, is an important factor affecting the biophysical properties and function of the skin barrier. Dry skin with a low hydration level is prone to having wrinkled, scaly, or rough properties, with the possible presence of cracking, reddening, or itching, and less flexibility compared to normal skin [53]. Skin hydration is also a crucial environmental factor enabling colonization by microorganisms in human skin. In agreement with previous studies, we observed a higher alpha diversity in dry skin; indeed, compared to sebaceous and moist sites, dry skin appears to be inhabited by a more mixed population [3]. In addition, we observed a significant difference in the phylum of *Firmicutes*, augmented in normal skin compared to dry skin. Furthermore, normal skin also showed high levels of spore-forming bacteria of the class *Clostridia*. On the contrary, the classes of *Alphaproteobacteria* and *Spirochetia* were significantly more abundant in dry skin with respect to normal groups.

Finally, we evaluated the host genetic elements that might influence the SM composition, using a candidate-gene approach. For this investigation, we chose representative traits affecting skin humidity and we associated the microbial abundance with host minor alleles on SNPs correlated with missense or non-sense mutation of those genes.

Intriguingly, we observed significant associations between microbiota members and SNPs, suggesting a negative correlation between collagen metabolism and ROS damage protection. In detail, a negative-correlation between *Negativicutes* and three *LTB4* SNPs was found. *LTB4* (latent transforming growth factor beta binding protein 4) is associated with the assembly of the extracellular matrix fibers, especially collagen, and glycosaminoglycans. Another correlation was observed between *Absconditabacteriales*_SR1 and on SNPs on *ALDH18A,* a gene involved in the biosynthesis of proline, ornithine, and arginine, precursors of collagen synthesis. Finally, we observed an association between *Negativicoccus* and one SNP of *PIK3R1* (phosphoinositide-3-kinase regulatory subunit 1), related to the cellular response to UV, epidermal growth factor receptor signaling pathway, and negative regulation of cell–matrix adhesion. Indeed, cellular response to UV generates ROS, which regulates gene expression related to collagen degradation and elastin accumulation. As modeling studies on the examined genes have not yet been carried out, we cannot check whether the individual gene variants affect the related three-dimensional protein structure. Therefore, it is noteworthy to point out again the potential links between SM components, ROS damage repair, and collagen biosynthesis. Moreover, to strengthen our findings, a recent study on cutaneous healing demonstrated the existence of a direct correlation between microbiome clades and collagen production, as a statistical correlation was observed between collagen, *Propionibacterium*, and *Staphylococcus* [54].

Although we explored the intrinsic characteristics of the human SM in the aspect of host genetics, our study is subject to some limits. First, there is a statistical limitation regarding the small sample sizes. Indeed, in genetic association studies, a sufficient number of samples is critical to detect causality between genes and phenotypes. However, we present the study as preliminary research with original but pioneering data that pave the way to further insights into a greater number of subjects, including different external and intrinsic factors that could impact the skin microbiota modulation. Furthermore, the collective size and the characteristics of the data set in microbiota analysis, alongside the possibility to also consider potential confounding factors in the model, lead to the need to increase the samples’ sizes to further confirm links between host genetic factors and the SM composition.

In addition, the sampling method used was able to collect only bacteria adhering to the upper skin layer. Moreover, we enrolled healthy women, where “healthy” referred only to the absence of overt dermatological pathologies. Additionally, our research was not a time-course study but compared groups of women at different ages, so it did not allow the study of the mechanisms of skin aging. Finally, there is an intrinsic microbiota variability between different subjects that we need to take into account.

## 5. Conclusions

Mutual correlation between the skin microbial community, skin aging, and genetic variant has not yet been sufficiently studied. Our explorative study suggests that the structure of the facial skin microbiome varies in women at different ages. Moreover, the functionality and associations of microbiota with host genetic factors may affect two aging-interrelated host pathways, such as ROS damage repair and the collagen metabolism. Given the importance of collagen in providing smoothness and elasticity to the skin and wound healing, it is crucial to obtain deeper knowledge about the abovementioned interactions, and to also investigate the effect of microbiome signaling and host genetic variation. Our data herein could pave the way for future studies characterizing the structure, function, and dynamics of the microbiome in the skin aging and regeneration processes. In addition, they may suggest the design of innovative and personalized therapeutic agents focused on host genomic targets, microbes, and their metabolites that contribute to skin healthcare, as well as provide a microbiological interpretation of the aging process.

## Figures and Tables

**Table 1:** Summary of the study population features.

Female Subjects	Codes	Age (Years)	Hydration Levels (A.U.) *	Daily Sun Exposition	Daily Use of Sunscreen	Smoke **	Skin Care Routine ***
1	A1	25	>45	No	No	Yes	No
2	A2	26	<45	Yes	No	No	Yes
3	A3	28	<45	Yes	Yes	Yes	No
4	A4	33	<45	No	No	No	Yes
5	A5	35	<45	Yes	No	No	Yes
6	B1	36	<45	No	No	Ex	No
7	B2	41	<45	Yes	Yes	Yes	Yes
8	B3	41	<45	No	No	No	No
9	B4	50	>45	No	No	No	Yes
10	B5	52	>45	Yes	Yes	No	Yes
11	C1	53	>45	Yes	Yes	No	Yes
12	C2	54	<45	No	Yes	Yes	Yes
13	C3	62	<45	Yes	Yes	No	Yes
14	C4	63	>45	No	No	No	Yes
15	C5	68	>45	No	No	No	No

* The level of skin humidity is expressed using arbitrary units (AU) as given by the device (Corneometer^®^CM825). Corneometry values greater than 45 AU indicate sufficiently moisturized skin (normal skin), while values less than 45 AU indicate dry skin. ** up to 5 cigarettes x day. “Ex” means “Ex-smoker”. *** Daily use of skin protection, prevention, cleansing, and moisturizing products.

**Table 2:** Summary of the taxonomic analysis of the obtained ASVs. Taxonomic assignment was performed using the SILVA 138 database.

	Total	Assigned Taxonomy	%
ASV	2647	2202	83.19%
Genus	506	487	96.24%
Family	221	216	97.74%
Order	117	115	98.29%
Class	43	42	97.67%
Phylum	22	21	95.45%

**Table 3:** Associations between the skin microbiota and SNPs.

Gene Symbol	SNPs	Chr	SNP chr Position	Minor Allele in Caucasian	Mutation Aminoacid [Nucleotide]	Associated Taxon	*p*-Value
*LTBP4*	rs1051303	19	41117806–41118306	G	T [ACC] > A [GCC]	*Negativicutes*	0.032
	rs1131620	19	41117619–41118119	G	T [ACT] > A [GCT]		
	rs2303729	19	41110819–41111319	A	I [ATA] > V [GTA]		
*ALDH18A1*	rs2275272	10	97387912–97388412	A	T [ACC] > I [ATC] (minus strand)	*Absconditabacteriales* (SR1)	0.045
*PIK3R1*	rs3730089	5	67587898–67588398	A	M [ATG] > I [ATA]	*Negativicoccus*	0.016

## Data Availability

Microbiota data was deposited in NCBI Gene Expression Omnibus under the accession number GSE225848 and the analysis script is available at https://github.com/matteoramazzotti/papers/tree/main/2021skin, (accessed on 20 February 2023).

## Supplementary Materials

The following supporting information can be downloaded at: https://www.mdpi.com/article/10.3390/biomedicines11030684/s1, Figure S1: Rarefaction analysis, performed to check the sample saturation after the reads discarded by DADA2. Table S1: Number of reads before and after the FASTQ processing. Table S2: Comparison of the abundance of single ASV. Table S3: Functional profiles associated with potentially expressed microbial genes in groups A and B. Table S4: Comparison of the abundance of single ASV. Table S5: Functional profiles associated with potentially expressed microbial genes in groups Dry and normal skin.
