# Supplementary material for: Facial Skin Microbiome: Aging-Related Changes and Exploratory Functional Associations with Host Genetic Factors, a Pilot Study
Source: Biomedicines. 2023 Feb 23;11(3):684. doi: 10.3390/biomedicines11030684 (PMC10045008; doi:10.3390/biomedicines11030684)
Supplement: Supplementary file 1 [file biomedicines-11-00684-s001.zip › biomedicines-2170432-supplementary.pdf]

Table S1. Number of reads before and after the FASTQ processing.

sample-id	input	filtered	denoised	merged	percentage of input merged	non-chimeric	percentage of input non-chimeric
A1	31838	23855	23594	23238	72.99	22884	71.88
A2	50115	37282	37050	36767	73.37	36370	72.57
A3	35495	27417	27204	26927	75.86	26717	75.27
A4	54113	43057	42911	42686	78.88	41596	76.87
A5	38042	30507	30371	30152	79.26	29341	77.13
B1	48303	37296	37108	36835	76.26	35881	74.28
B2	44514	36254	36143	35996	80.86	35533	79.82
B3	360535	298217	297441	296096	82.13	292288	81.07
B4	20647	15331	15104	14701	71.2	14389	69.69
B5	111386	89854	89321	87987	78.99	85011	76.32
C1	101250	81896	81540	80707	79.71	77247	76.29
C2	95544	77079	76958	76775	80.36	75684	79.21
C3	34179	27667	27443	27212	79.62	26855	78.57
C4	114346	89608	89395	88856	77.71	87437	76.47
C5	78973	61830	61643	61337	77.67	59857	75.79

Table S2. Comparison of the abundance of single ASV.

Num*	Den*	Taxonomic level	Basemean	Log2FC	lfcSE**	stat	Adjusted p-value	Taxon
B	A	Phylum	33.933	21.139	2.751	7.684	2.16E-13	*Campilobacterota*
C	A	Phylum	33.933	20.948	2.751	7.614	3.73E-13	*Campilobacterota*
B	A	Phylum	11.135	18.921	3.580	5.285	8.81E-07	*Spirochaetota*
C	A	Phylum	11.135	20.032	3.577	5.600	1.50E-07	*Spirochaetota*
B	A	Class	17806.818	-1.475	0.541	-2.726	0.043323	*Actinobacteria*
C	B	Class	17806.818	1.704	0.541	3.148	0.022155	*Actinobacteria*
B	A	Class	46.616	20.843	3.249	6.415	3.80E-09	*Campylobacteria*
C	A	Class	46.616	21.668	3.249	6.669	6.93E-10	*Campylobacteria*
B	A	Class	511.070	2.970	0.835	3.559	0.003353	*Negativicutes*

C	B	Class	511.070	-2.996	0.834	-3.593	0.008831	*Negativicutes*
B	A	Order	17.218	19.827	2.927	6.773	4.77E-10	*Absconditabacteriales_(SR1)*
C	A	Order	17.218	20.123	2.928	6.873	2.17E-10	*Absconditabacteriales_(SR1)*
C	B	Order	3606.273	3.408	1.004	3.393	0.023892	*Corynebacteriales*
B	A	Order	6.885	18.797	3.360	5.594	5.11E-07	*Cytophagales*
C	A	Order	6.885	18.785	3.363	5.586	4.01E-07	*Cytophagales*
C	A	Order	13.499	-22.045	3.654	-6.033	3.70E-08	*Exiguobacterales*
C	B	Order	13.499	-21.960	3.653	-6.012	1.27E-07	*Exiguobacterales*
B	A	Family	6.172	18.977	3.378	5.618	1.19E-06	*Nocardioidaceae*
C	A	Family	6.172	18.393	3.384	5.436	1.83E-06	*Nocardioidaceae*
B	A	Genus	51.023	21.533	3.263	6.599	1.08E-08	*Abiotrophia*
C	A	Genus	51.023	21.609	3.264	6.621	6.43E-09	*Abiotrophia*
B	A	Genus	16.694	20.540	3.452	5.950	2.33E-07	*Flavobacterium*
C	A	Genus	16.694	19.281	3.458	5.575	1.13E-06	*Flavobacterium*
B	A	Genus	53.188	13.407	3.738	3.587	0.017489	*Lactococcus*
C	A	Genus	53.188	22.791	3.650	6.243	3.72E-08	*Lactococcus*
B	A	Genus	14.211	18.443	3.657	5.044	2.98E-05	*Treponema*
C	A	Genus	14.211	20.336	3.653	5.567	1.13E-06	*Treponema*

*"Num "and the" Den "are standard names used for DESeq2, they means respectively Numerator "vs". Denominator ", representing the two compared groups. ** Log2 Fold Change Standard Error.

Table S3. Functional profiles associated with potentially expressed microbial genes in groups A and B.

KO entry	EC number	Gene symbol	Description	Different class	LDA Score (log10)
K01270	3.4.13	*pepD*	dipeptidase D	B	2.041494095
K12267	1.8.4.11 / 1.8.4.12	*msrAB*	peptide methionine sulfoxide reductase	B	2.035527
K05366	2.4.1.129 / 3.4.16.4	*mrcA*	penicillin-binding protein	B	2.098276119
K01647	2.3.3.1	*CS, gltA*	citrate synthase	A	2.091586362
K01895	6.2.1.1	*ACSS, acs*	acetyl-CoA synthetase	A	2.354463699
K03750	2.10.1.1	*moeA*	molybdopterin molybdotransferase	A	2.153599221
K02221		*yggT*	uncharacterized protein	A	2.113230439
K00241		*sdhC, frdC*	succinate dehydrogenase / fumarate reductase	A	2.067092941
K00240	1.3.5.1 / 1.3.5.4	*sdhB, frdB*	succinate dehydrogenase / fumarate reductase	A	2.048086623

EC= enzymatic class, if missing it means that there are no correspondence between the two compared databases

Table S4. Comparison of the abundance of single ASV.

Num*	Den*	Taxonomic level	baseMean	log2FC**	lfcSE	stat	Adjusted p-value	Taxon
Dry Skin	Normal	Phylum	15051.04	-1.81	0.58	-3.13	1.50E-02	*Firmicutes*
Dry Skin	Normal	Class	16.31	23.76	3.02	7.86	9.78E-14	*Spirochaetia*
Dry Skin	Normal	Class	11399.57	-1.54	0.46	-3.31	7.88E-03	*Bacilli*
Dry Skin	Normal	Class	425.21	1.81	0.63	2.90	2.35E-02	*Alphaproteobacteria*
Dry Skin	Normal	Class	3624.56	-2.53	0.90	-2.81	2.48E-02	*Clostridia*
Dry Skin	Normal	Genus	15.46	-21.81	3.06	-7.12	2.50E-10	*Negativicoccus*
Dry Skin	Normal	Genus	1120.43	-5.62	1.56	-3.61	3.60E-02	*Peptoniphilus*

*“Num "and the" Den "are the standard names used for DESeq2 uses, they means respectively Numerator "vs". Denominator ", representing the two compared groups. ** Log2 Fold Change Standard Error.

Table S5. Functional profiles associated with potentially expressed microbial genes in groups Dry and normal skin.

KO Entry	EC number	Gene symbol	Description	Different Class	LDA Score (log10)
K00116	1.1.5.4	*Mqo*	malate dehydrogenase	hydratation < 45	2.171081
K03311		*TC.LIVCS*	cation transporter	hydratation < 45	2.022262
K01258	3.4.11.4	*pepT*	tripeptide peptidase	hydratation < 45	2.00751
K03100	3.4.21.89	*lepB*	enzyme signal peptidase	hydratation < 45	2.04445
K07491		*rayT*	Reparation associated tyrosine transposase	hydratation < 45	2.114414
K07498		*putative transposase*	putative transposase	hydratation < 45	2.275746
K00712	2.4.1.52	*tagE*	alpha-glucosyltransferase	hydratation < 45	2.082537
K03406		*Mcp*	chemotaxis protein	hydratation > 45	2.15397
K03088		*rpoE*	RNA polymerase sigma-70 factor	hydratation > 45	2.258803
K03496		*parA, soj*	chromosome partitioning protein	hydratation > 45	2.091799

EC= enzymatic class, if missing it means that there are no correspondence between the two compared databases

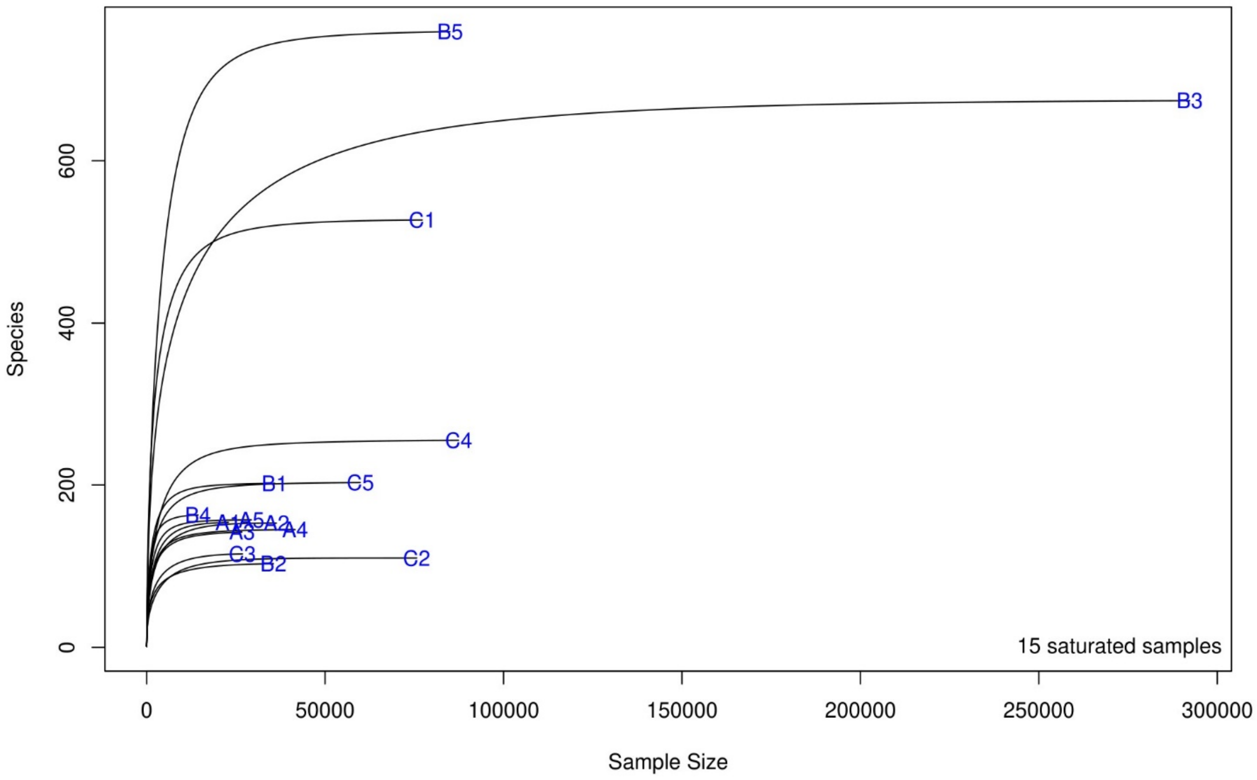

Figure S1. Rarefaction analysis, performed to check the sample saturation after the reads discarded by DADA2.
